# Different renal phenotypes in related adult males with Fabry disease with the same classic genotype

**DOI:** 10.1002/mgg3.292

**Published:** 2017-05-08

**Authors:** Renzo Mignani, Mariarita Moschella, Giovanna Cenacchi, Ilaria Donati, Marta Flachi, Daniela Grimaldi, Davide Cerretani, Paola De Giovanni, Marcello Montevecchi, Angelo Rigotti, Alessandro Ravasio

**Affiliations:** ^1^ Nephrology and Dialysis Department Infermi Hospital Rimini Italy; ^2^ Pathology and Ultrastructural Anatomy S. Orsola University Hospital Bologna Italy; ^3^ Genetic Department Infermi Hospital Rimini Italy; ^4^ Neurology Department Infermi Hospital Rimini Italy

**Keywords:** Fabry disease, genotype, mutation, phenotype, proteinuria, variability

## Abstract

**Background:**

Fabry disease related patients with classical mutation usually exhibit similar severe phenotype especially concerning renal manifestation.

**Methods:**

A dry blood spot screening (DBS) and the DNA analysis has been performed in a 48‐year‐old man (Patient 1) because of paresthesia.

**Results:**

The DBS revealed absent leukocyte *α*‐Gal A enzyme activity while DNA analysis identified the I354K mutation. Serum creatinine and e‐GFR were in normal range and also albuminuria and proteinuria were absent. The brain MRI showed ischemic lesions and a diffuse focus of gliosis in the white matter, while the echocardiogram showed a left ventricular hypertrophy. The renal biopsy performed in the case index showed a massive deposition of zebra bodies. By a familiar investigation, it was recognized that his brother (Patient 2) died 2 years before from sudden death syndrome at the age of 49. He had suffered sporadic and undiagnosed pain at the extremities, a prior cataract, bilateral neurosensorial hearing loss and left ventricular hypertrophy on Echocardiogram. His previous laboratory examinations revealed a normal serum creatinine and the absence of proteinuria. Pedigree analysis of the brothers revealed a high disease burden among family members, with an affected cousin (Patient 3) who progressed early to end‐stage renal disease (ESRD) that required renal transplantation.

**Conclusions:**

Here we describe the clinical history of three adult male members of the same family with the same genotype who manifested different presentation and progression of the disease, particularly concerning the renal involvement.

## Introduction

Fabry disease (OMIM 301500) is a rare X‐linked disorder resulting from deficient activity of the lysosomal enzyme *α*‐galactosidase A, due to mutations in the α galactosidase A (GLA) gene localized in the X chromosomal region q22.1. The enzymatic defect leads to a progressive accumulation of glycosphingolipids in several fluids, cells, and in most visceral tissues including kidney, heart, brain, connective tissue, peripheral nerves, and vascular endothelium (Desnick et al. 2001). In the classic form of the disease, the symptoms start in childhood with pain and paresthesia, while renal manifestations with albuminuria and proteinuria manifest in adolescence. During the second‐to‐third decades of life, a hypertrophic cardiomyopathy occurs, while the renal involvement progresses to chronic renal failure leading to early end‐stage renal disease (ESRD) that requires dialysis and transplantation. In male patients with classic Fabry disease, the leukocyte *α*‐Gal A activity is generally absent or <1%. In 13% of patients (Spada et al. 2006), the leukocyte *α*‐Gal A activity is above 10% and symptoms onset occurs in the fourth‐to‐ fifth decades of life. In these late‐onset phenotypes, the progression and the severity of the disease are milder and symptoms are mainly represented by cardiac involvement with arrhythmias and hypertrophic cardiomyopathy, while other signs and symptoms of the disease are mild or absent (Hsu et al. 2016). The diagnosis in male patients is based on the detection of a low or absent plasma or leukocyte *α*‐Gal A activity that must be then confirmed by DNA analysis. In females the diagnosis is exclusively based on gene sequencing, since they can show *α*‐Gal A activity ranging from normal to very low levels due to the lyonization phenomenon. Moreover, female patients experience an extreme variability in phenotype ranging from asymptomatic to classic phenotype (Echevarria et al. 2016). In Fabry disease, about 770 mutations in the α galactosidase A (GLA) gene have been described so far (Schiffmann and Ries 2016). Although, some of these are associated with a classic phenotype and others to a late‐onset phenotype, genotype/phenotype correlation is still controversial. Across the same family, variability in the phenotype has been often documented between female members but also among male patients with late‐onset mutations, and between male patients with severe or mild renal involvement (Redonnet‐Vernhet et al. 1996; Veronik et al. 2004; Rigoldi et al. 2014; Pan et al. 2016). By contrast, a different renal phenotype between adult male patients belonging to the same family, but without any clinical sign of renal damage, has not been described yet. Here, we describe the clinical history of three adult male members of the same family: two of them with the same genotype and one with a high probability of having the same genotype. All of them manifested different presentation and progression of the disease, particularly concerning the renal involvement.

## Case Report

**Figure 1:**
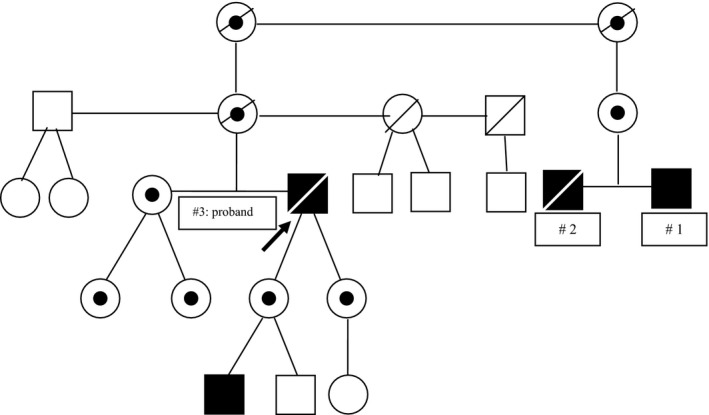
Pedigree of the family**.** 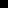 Fabry male patient. 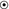 Heterozygous female. 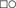 Unaffected patients. 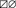 Deceased patients.

Here, we describe the clinical history of three adult male members belonging to the same family affected by the missense mutation c.1061T>A p.Ile354Lys or I354K (Fig. 1). Patient 1 is a 48‐year‐old‐man patient recently diagnosed with Fabry disease with normal renal function without proteinuria; his brother (Patient 2) died at the age of 49 from sudden death syndrome with normal renal function without proteinuria; their cousin (Patient 3, proband of the family) was affected by ESRD and died at the age of 59 after kidney transplantation.

### Patient 1

**Figure 2:**
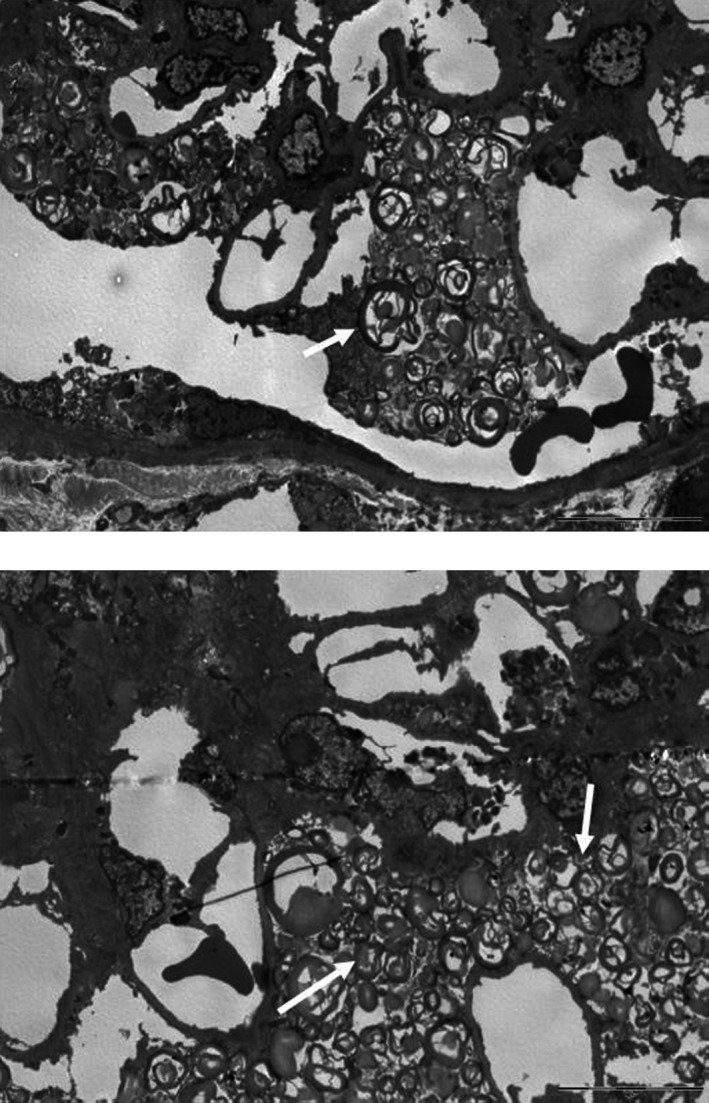
Massive presence of zebra bodies into podocytes and mesangial matrix of the glomerulus.

MaZ is a 48 year‐old‐man, healthy until 2 years ago. In 2014, he was admitted to hospital because of paresthesia and hyposthenia on the right‐half of his body. The biochemical evaluation showed a serum creatinine level of 0.8 mg/dL with an e‐GFR of 78 mL/min/1.73 m^2^. The brain MRI demonstrated ischemic lesions at the left pontine side and in the left radial crown, and a diffuse focus of gliosis in the white matter. The electrocardiogram showed a PR interval of 125 msec and in the echocardiogram (ECHO) a left ventricular hypertrophy was present with a posterior wall thickness (PWT) of 12 mm and a interventricular septum thickness (IVS) of 18 mm. Due to the persistence of pain, 2 years later, a dry blood spot screening was performed revealing absent *α*‐Gal A enzyme activity, and DNA analysis identified the variant p.Ile354Lys. Subsequent evaluation demonstrated the absence of angiokeratomas, the presence of cornea verticillata and a moderate bilateral hearing loss on audiometry. The biochemical investigation showed serum creatinine of 0.7 mg/dL, e‐GFR of 96 mL/min/1.73 m^2^, proteinuria 80 mg/day, and albuminuria 3 mg/mmol. Due to this atypical presentation of the disease, and after receiving informed consent, a renal biopsy was performed. The major findings from light microscopy involve the podocytes, which appeared markedly enlarged, with bubbly, clear, foamy cytoplasm. Although the glomeruli were normocellular, the swollen podocytes had a tendency to narrow the urinary space. Similar lipid vacuoles were detected in tubular cells, vascular myocytes, and endothelial cells. By electron microscopy, diagnostic myelin figures were identified diffusely in the cytoplasm of podocytes, with variable but less conspicuous involvement of mesangial cells, glomerular endothelial cells, tubular epithelial cells (particularly distal), arterial myocytes and endothelial cells (Fig. 2). Immunofluorescence staining was negative for deposits. After the conclusion of disease staging, the patient started enzyme replacement therapy (ERT) with agalsidase beta at a standard dose.

### Patient 2 (brother)

MiZ was a healthy 44‐year‐old‐man when he underwent an operation for a posterior right cataract. Two years later, he began to complain of pain and paresthesia at the upper extremities and a mild bilateral neurosensorial hearing loss. The CT scan did not demonstrate any white matter or vascular lesions in the brain. At the age of 47, further cardiologic examinations with echocardiogram demonstrated the presence of hypertrophic cardiomyopathy with a severe interventricular septum thickness of 17 mm, while biochemical exams showed a serum creatinine of 0.7 mg/dL, with an e‐GFR of 89 mL/min/1.73 m^2^. Both albuminuria and proteinuria were in the normal range (2.5 mg/mmol and 90 mg/day, respectively). In 2011, at the age of 49, he died of sudden death syndrome; the autopsy documented a massive intracerebral hemorrhage as the cause of death.

### Patient 3 (proband)

WM was a 28‐year‐old male when he was referred to our department for mild proteinuria in 1981. The kidney biopsy demonstrated typical Fabry disease nephropathy under optical and ultrastructural analysis. The leukocyte *α*‐Gal A enzyme activity was totally absent, while the DNA analysis identified the variant p.Ile354Lys. The systemic evaluation of the patient demonstrated the presence of cornea verticillata, a posterior bilateral cataract, some angiokeratomas in the scrotum, a mild bilateral neurosensorial hearing loss, and a moderate IVS on echocardiogram. In 1992 the hemodialysis treatment was started, and 1 year later he received a successful cadaveric kidney transplant. Immunosuppression was based on steroids, cyclosporine and azathioprine; on discharge the serum creatinine was 2 mg/dL and proteinuria was absent. Afterward, the graft function remained stable over several years, while a progressive Fabry cardiomyopathy with chronic atrial fibrillation occurred. In 2001, he started enzyme replacement therapy (ERT) with a standard dose of agalsidase beta that was only reduced during the shortage period. In 2012, he died of chronic heart failure with a functional renal transplant.

## Discussion

We report here the unusual history of two adult male patients with Fabry disease who have the same missense mutation p.Ile354Lys because they belonged to the same family. In addition, the brother of one of these males had a medical history that raises a high suspicion of also being affected by the same mutation. All three males exhibited different phenotypes of the disease with regard to the renal manifestations. In particular, the proband of the family (Patient 3) was diagnosed with Fabry disease about 30 years ago at the age of 28, with proteinuria and renal failure that progressed to ESRD which required renal transplantation (Mignani et al. 2008). He also developed a severe cardiomyopathy that led to heart failure and death at the age of 59. In both affected daughters and nieces of this patient (Patient 3), the disease manifested early with albuminuria and proteinuria leading to an early start of ERT (Fig. 1). The severity of clinical manifestation in the proband, together with the early onset in female siblings, prompted us to consider this mutation as classic. After the diagnosis of the proband, we completed family screening with his reassurance, discovering a further six members affected by the disease (Fig. 1). However, only subsequently we found an affected 1st degree cousin (Patient 1) diagnosed with Fabry disease by a neurologist without any clinical signs of renal involvement, but with typical Fabry nephropathy on biopsy. Completing the family history, we recognized that his brother (Patient 2), who died 5 years before from a hemorrhagic stroke, had also probably been affected by Fabry disease, since he had been suffering from several symptoms of the disease (pain, posterior cataract, bilateral hearing loss, hypertrophic cardiomyopathy) but without any sign of renal damage. What surprised us was the great difference in the severity of renal manifestations between these three adult males, with the proband affected by ESRD and two cousins with normal renal function and without proteinuria or albuminuria. The origin of phenotype variability in Fabry disease is quite controversial. Phenotype variability has already been described many times in female patients, and also between monozygotic twins with the same genotype, due to the Lyonization phenomenon (Redonnet‐Vernhet et al. 1996; Echevarria et al. 2016). In affected male children and adolescents who are related, it is common to observe the onset of pain, parestesias, albuminuria, or proteinuria at different ages (Schiffmann and Ries 2016). These observations do not strictly reflect the presence of different phenotypes, but rather just the expression at different ages of disease signs and symptoms onset. A high interfamilial variability has been previously documented in a Slovenian family. In this report, however, together with males on ESRD, some other family members with normal renal function had already presented renal clinical signs with overt proteinuria (Veronik et al. 2004). More recently, in four Italian pedigrees, several cases of interfamilial variability have been documented, but some males already had albuminuria and their age was not indicated (Rigoldi et al. 2014). Renal variability has been documented recently in a large cohort of Chinese patients, and in particular in a family with the R301Q mutation, but this is now considered to be a late‐onset variant (Pan et al. 2016).

Our findings confirm previous data on intrafamilial variability in hemizygous males with classical Fabry disease. However, in the family members presented here, two of the three males with the same mutation (and absent *α*‐Gal A enzyme activity) had no clinical signs of renal involvement, despite the renal biopsy in one of them showed a massive deposition of substrate. To our knowledge, this is the first case of related males having no clinical signs of renal involvement at this age. The reason for the phenotypic variability among male members of the same family with the same classic mutation is still not clear. Several hypotheses have been suggested such as the presence of concomitant environmental factors, or the action of modifier genes that could influence the phenotype of Fabry disease. The consequence of this variability may be the lack of reliability and prognostic value of genotype, in particular of classic genotype. Often, it happens that clinicians start ERT in affected asymptomatic children only because they are the offspring of adults with classic mutations which are responsible for severe disease burden. In the majority of cases, this approach is right, but we should not exclude the possibility of a different phenotypical outcome. Therefore, this report should prompt us to be careful in the genetic counseling of family members, and in particular when predicting the disease course and consequently deciding if and when to start ERT in Fabry disease patients.

## Ethical Approval

All procedures performed in the studies involving human participants were in accordance with the ethical standards of the institutional and/or national research committee, and with the 1964 Helsinki declaration and its later amendments or comparable ethical standards.

## Conflict of Interest

The authors declare that they have no conflicts of interest.
